# Development of a Modified Global Physical Activity Questionnaire and Its Construct Validity among Adults in Kerala, India

**DOI:** 10.3390/diabetology4020020

**Published:** 2023-06-20

**Authors:** Thirunavukkarasu Sathish, Elezebeth Mathews

**Affiliations:** 1 Department of Family and Preventive Medicine, School of Medicine, Emory University, Atlanta, GA 30322, USA; 2 Emory Global Diabetes Research Center, Woodruff Health Sciences Center, Emory University, Atlanta, GA 30322, USA; 3 Department of Public Health and Community Medicine, Central University of Kerala, Kasaragod 671320, Kerala, India

**Keywords:** physical activity, GPAQ, validity, self-reports, chronic diseases

## Abstract

There is a need for a modified Global Physical Activity Questionnaire (GPAQ) to estimate physical activity levels more accurately in populations. We aimed to develop a modified GPAQ and examine its construct validity among adults in Kerala, India. We incorporated locality-specific, metabolic equivalent task (MET)-based activities into the original GPAQ and administered this modified GPAQ among randomly selected 451 individuals (age ≥ 20 years) residing in the Trivandrum district of Kerala. Construct validity of the modified GPAQ was assessed using generalized linear models by examining the association of total moderate-to-vigorous physical activity (MVPA) MET-minutes per week with clinical measures. The mean age of participants was 45.4 (SD: 14.1) years, and 52.6% were female. Increasing total MVPA MET-minutes per week was associated with decreasing weight (β = *−*0.011 kg, 95% CI: −0.020, −0.002), waist circumference (β = −0.013 cm, 95% CI: −0.023, −0.004), and systolic blood pressure (β = −0.009 mmHg, 95% CI: −0.015, −0.002), independent of age, sex, education, occupation, current smoking, current alcohol use, and fruit and vegetable intake. The validity coefficients and associations between total MVPA MET-minutes per week and theoretical constructs of physical activity agreed with those predicted, providing evidence of construct validity for the modified GPAQ.

## 1 Introduction

Physical inactivity is considered a pandemic worldwide [1] and is an independent risk factor for type 2 diabetes, cardiovascular diseases, and breast and colon cancers [2]. Therefore, accurate measures of physical activity are essential to assess the prevalence and secular trends of physical activity in populations [3] and implement effective non-communicable disease prevention programs [4]. Although, there are several standardized objective techniques for measuring physical activity, their use is limited in population-based studies, especially in low- and middle-income countries (LMICs) such as India, due to cost constraints. Therefore, assessing self-reported physical activity through questionnaires is recognized as the best compromise [5].

The Global Physical Activity Questionnaire (GPAQ) was developed by the World Health Organization (WHO) for the surveillance of physical activity levels within countries and to compare physical activity estimates across countries [6,7]. The GPAQ is accompanied by “show cards” with examples of moderate- to-vigorous (MVPA) physical activity. Respondents identify activities listed in the show cards based on the intensity of the activity and report the frequency and duration of those activities performed in a “typical” week. The GPAQ recommends including locality-specific activities in the show cards, with the intensity (moderate or vigorous) of activities determined based on “self-perception” by individuals rather than “metabolic equivalent task (MET)” values obtained from laboratory or field experiments that measure the oxygen cost of specific activities [7]. Studies show that adults often consider “low-intensity” activity as “moderate” and “moderate-intensity” activity as “vigorous” [8,9]. Consequently, the GPAQ overestimates physical activity, as shown by studies conducted in India and other countries [10–12]. Further, in the Nine Country study by Bull FC et al. [13], India was excluded from all reliability analyses (due to errors in the data), and only criterion validity against pedometers was measured. On a sample of 234 adults, Spearman’s rho for criterion validity was poor (0.35) for total physical activity [13]. The construct validity of the GPAQ was not established in this study or in other studies from India. ‘Construct validity refers to the degree to which inferences can legitimately be made from the operationalizations in your study to the theoretical constructs on which those operationalizations were based’ [14]. The construct validity of a measure can be established by correlating it with a number of other measures and arguing from the pattern of correlations that the measure is associated with these variables in theoretically predictable ways [15].

In this study, we aimed to develop a modified GPAQ by incorporating locality-specific activities, which were MET-based, into the original GPAQ in an Indian population. We also examined the construct validity of this modified GPAQ by examining the association of total MET-minutes per week with clinical measures.

## 2 Materials and Methods

### 2.1 Study Design and Participants

This is a secondary analysis of a cohort study established in the Indian state of Kerala to primarily assess the longitudinal changes in the burden of risk factors for non-communicable diseases (NCDs) over seven years. The original study design is described in detail elsewhere [16,17]. Briefly, between 2003 and 2006, a large cross-sectional survey of risk factors for NCDs was conducted among 2510 randomly selected individuals (aged 15 to 64 years) from rural areas of Trivandrum district in Kerala state, India [18]. The sample size was calculated using the means of the NCD risk factors, as per the World Health Organization (WHO) STEPwise approach to surveillance (STEPS) protocol [6]. From this sample of 2510 individuals, 495 were selected using a systematic random sampling method, of which 452 (91.3%) were followed up in 2010 [16,17]. The reasons for the loss of follow-up were: death (n = 17), relocation (n = 10), decline to participate (n = 8), pregnancy (n = 4), and not traceable (n = 4). One pregnant woman at baseline was excluded, leaving 451 participants for the present analysis.

### 2.2 Development and Administration of the Modified GPAQ

We developed the modified GPAQ in two steps. Firstly, we identified activities that were commonly performed at work, travel, and leisure by males and females (aged *≥* 30 years) residing in local neighborhoods. This was performed through in-depth interviews with five males and five females, focus group discussions with two males and four females, and direct observations by trained research staff who live in the communities where the original cohort study was conducted. Direct observations were unstructured, with the staff recording their observations as field notes, and covert (individuals living in the neighborhoods were unaware of the purpose of the observation). The MET value of the local activities was obtained from the updated 2011 Adult Compendium of Physical Activities [7]. A MET value of 4 was assigned to moderate activity and 8 to vigorous activity, according to the GPAQ analysis guide [6]. One MET is the amount of oxygen intake while sitting at rest and is approximately equal to 3.5 mL O_2_ kg^−1^ min^−1^ [7]. For activities with no MET value available, the opinion of experts in the area of physical activity was ascertained to categorize the activities as moderate or vigorous. Secondly, we included the identified activities in the original GPAQ and asked for the number of days that particular activity was performed on a typical week and the time spent doing that activity on a typical day. These questions replaced the following original questions in the GPAQ: “*In a typical week, on how many days do you do vigorous-intensity activities as part of your work?*” and “*How much time do you spend doing vigorous-intensity activities at work on a typical day?*” See Supplementary Material (S1) for the modified GPAQ. The modified GPAQ was developed in English and translated into Malayalam (the local language), which was pilot tested on 15 individuals (10 women and 5 men) identified from the local community. Pilot testing showed that the modified GPAQ was feasible to implement as it was self-explanatory, the activities listed in the show cards were comprehensive, and the average time taken to administer the questionnaire to one participant was 15 min. For the main study, trained research staff, who were identified from the local community itself, administered the modified GPAQ to participants in their households.

### 2.3 Total MVPA MET-minutes per Week

We calculated total moderate-to-vigorous physical activity (MVPA) MET-minutes per week from the modified GPAQ as follows: If Mr. A carries heavy loads (vigorous activity) for two days a week for 45 min a day during work (8 × 2 × 45 = 720 MET-minutes per week), walks (moderate activity) for five days a week for 30 min a day (4 × 5 × 30 = 600 MET-minutes per week), and plays football (vigorous activity) for five days a week for 30 min a day (4 × 5 × 30 = 600 MET-minutes per week), the total MVPA MET-minutes per week for this individual is 1920. Using total MVPA MET-minutes per week, we categorized the physical activity levels of participants as low (<600 MET-min per week), moderate (600–2999 MET-min per week), or high (*≥*3000 MET-min per week), as per the GPAQ analysis guide [6].

### 2.4 Data Collection and Variables

Trained research staff interviewed participants using the WHO STEPS questionnaire [6] to collect data on age, sex, years of schooling, occupation (skilled or unskilled labor, homemaker, retired, student, or unemployed), smoking, alcohol use, and fruit and vegetable intake. Current smokers were defined as individuals who had smoked any tobacco (cigarettes, bidis [hand-rolled cigarettes], cigars, or hookahs [water pipes]) in the last 30 days [6]. Current alcohol users were those who had consumed at least one standard drink of alcohol (30 mL of spirits, 285 mL of beer, or 120 mL of wine) in the last 30 days [6]. Consumption of <400 g (<5 servings) of fruits and vegetables per day was considered low fruit and vegetable intake [6]. We measured weight, waist circumference, and blood pressure (BP) using standard tools based on the WHO STEPS protocol [6]. Weight was measured using a TANITA body composition analyzer (model SC330) while the participant was standing still without footwear, with one foot on each side of the scale, facing forward, and arms at their sides. The waist circumference was measured using a non-stretchable Seca measuring tape over bare skin at the midpoint between the lower margin of the last palpable rib and the top of the iliac crest. The BP was measured using an Omron digital automatic BP monitor on the right arm in a seated position after having rested for at least five minutes. Two BP readings were obtained initially, and a third reading was taken if there was a difference of more than 10 mmHg in systolic or diastolic BP between the initial two readings. Accordingly, the average of two or more readings was considered for the analyses.

### 2.5 Statistical Analysis

Data were analyzed using Stata version 17.0 (StataCorp LP, College Station, TX, USA). Continuous variables are presented as mean (standard deviation, SD) or median (interquartile range [IQR]), and categorical variables as n (%). The construct validity of the modified GPAQ was examined using generalized linear models (GLM) [19] to examine whether total MVPA MET-minutes per week were predictive of clinical measures, including weight, waist circumference, systolic BP, and diastolic BP. These variables were chosen as “theoretical constructs” because physical activity has been shown to be predictive of them in several cross-sectional as well as prospective cohort studies [20–22]. Given the skewed nature of total MVPA MET-minutes per week, the GLM models were fitted with a gamma family and a log-link function [19]. Both univariate and multivariate analyses were performed. The multivariate models were adjusted for potential confounders, including age, sex, years of schooling, occupation, fruit and vegetable intake, current smoking, and current alcohol use. Collinearity between independent variables was checked with the ‘*coldiag*’ command in Stata, and the condition number was less than 30, indicating that there was no significant collinearity between variables [23]. A two-tailed *p* < 0.05 was considered to be significantly significant for analyses.

## 3 Results

Table 1 shows the characteristics of study participants. The mean age was 45.4 (SD: 14.1) years, slightly more than half (52.6%) were female, and the mean years of schooling were 8.6 (SD: 4.3). The majority (55.2%) of participants were employed, followed by homemakers (36.1%) and retired, student, or unemployed (8.7%). Nearly a quarter (21.1%) had low levels of physical activity, while 4.9% were moderately active, and almost three-fourths (74.1%) were highly active.

Table 2 shows the median MET-minutes per week by domains of the GPAQ. The median total MVPA MET-minutes per week was 12,180 (IQR: 2640–40,320), with most of which from work-related activities (72.4%, 8820/12,180). The median MET-minutes per week was 0 in the leisure domain, as only 10.6% of participants were engaged in leisure-time activities.

The results of the univariate analysis for the association between total MVPA MET-minutes per week and various clinical measures are given in Table 3. The total MVPA MET-minutes per week were negatively associated with mean age (β = −0.02, 95% CI: −0.03 to 0.01), female sex (β = −0.58, 95% CI: −0.78 to −0.37), mean years of schooling (β = −0.036, 95% CI: −0.068 to −0.004), occupation (homemaker: β = −1.11, 95% CI: −1.34 to −0.89; retired/student/unemployed: β = *−*2.50, 95% CI: −2.88 to −2.11; paid labor as reference), current alcohol use (β = −0.47, 95% CI: *−*0.47, −0.71 to −0.24), mean weight (β = −0.013, 95% CI: −0.023 to −0.004), mean waist circumference (β = −0.025, 95% CI: −0.034 to −0.016), and positively associated with current smoking (β = 0.56, 95% CI: 0.26 to 0.86). Low fruit and vegetable intake (<5 servings/day) and diastolic BP were not significantly associated with the total MVPA MET-minutes per week.

The results of multivariate analysis for the association between total MVPA MET-minutes per week and various clinical measures are given in Table 4. Increasing total MVPA MET-minutes per week was significantly associated with decreasing weight (β = −0.010 kg, 95% CI: −0.019, −0.001), waist circumference (β = −0.013 cm, 95% CI −0.023 to −0.004), and systolic BP (β = −0.009 mmHg, 95% CI: −0.016, −0.003), independent of age, sex, education, occupation, current smoking, current alcohol use, and fruit and vegetable intake.

## 4 Discussion

In this study, we developed a culturally appropriate, locality-specific, and MET-based modified GPAQ and assessed its construct validity in a sample of the general population residing in Kerala state, India. The total MVPA MET-minutes per week estimated from the modified GPAQ were significantly and negatively associated with clinical measures, including weight, waist circumference, and systolic BP, after accounting for potential confounders, thereby establishing its construct validity.

Physical inactivity is an independent risk factor for several NCDs. Thus, obtaining precise estimates of physical activity in populations is imperative for designing NCD prevention and management programs [1]. We developed a modified GPAQ by incorporating locality-specific, MET-based activities into the original GPAQ. This way, the original GPAQ was modified to be more culturally appropriate and evidence-based in measuring physical activity levels of the general population. In our previous study, we validated this modified GPAQ against accelerometers in a sample of 47 women (aged 18–64 years) residing in the same neighborhoods as our study population in the Trivandrum district of Kerala [24]. We found that for total MVPA, the correlation between the modified GPAQ and accelerometer was moderate (Spearman’s rho = 0.69), and the level of agreement between the two tools was moderately high with an intra-class correlation coefficient (ICC) of 0.78. These figures are higher than those reported using the original GPAQ in the nine-country study conducted among Indian adults [13] and many other studies globally [25–28]. In addition, the level of overestimation of physical activity levels by this modified GPAQ was much lower than that found in other studies with the original GPAQ [29].

This is one of the first studies to develop a modified GPAQ and assess its construct validity among the general population in India. Further, the study was conducted in Kerala, which has a high burden of type 2 diabetes and other cardiometabolic risk factors and is supposedly the harbinger for the rest of India in terms of the burden of NCDs [16,17,30], as the state is in the most advanced stage of epidemiological transition [31]. Thus, Kerala is an ideal place in India to develop a modified GPAQ and assess its psychometric properties. Finally, the methodology that was used to develop the modified GPAQ could be adopted by other LMICs in which the activities are culturally diverse. However, there are certain limitations to using this modified GPAQ. First, elderly individuals, particularly women, were not able to recall the time spent on certain activities, even though they were performed regularly. Second, the MET values of activities were based on Western standards, which may be different for South Asian populations. Future research is required to estimate the MET value of region-specific activities. Finally, the average time to administer the modified GPAQ (~15 min) is slightly longer than that of the original GPAQ (~10 min).

## 5 Conclusions

To conclude, the association between total MVPA MET-minutes per week and theoretical constructs of physical activity agreed with those predicted, providing evidence of construct validity for the modified GPAQ. Further research should focus on studying the association between physical activity levels measured by this modified GPAQ and the risk of developing type 2 diabetes and other cardiometabolic risk factors in India.

## Supplementary Material

Supplementary Materials: The following supporting information can be downloaded at: https://www.mdpi.com/article/10.3390/diabetology4020020/s1, Table S1: Modified GPAQ.

Supplementary Materials

## Figures and Tables

**Table 1:** Characteristics of study participants.

Variable	N = 451
Age (years)	45.4 (14.1)
Sex (female)	237 (52.6)
Years of schooling	8.6 (4.3)
**Occupation**	
Employed *	249 (55.2)
Homemaker	163 (36.1)
Retired/student/unemployed	39 (8.7)
Current smoking (yes)	72 (16.0)
Current alcohol use (yes)	166 (36.8)
<5 servings of fruit and vegetables/day	303 (67.2)
Weight (kg)	59.5 (11.3)
Waist circumference (cm)	88.5 (11.4)
Systolic blood pressure (mmHg)	133.0 (20.5)
Diastolic blood pressure (mmHg)	81.1 (10.2)
**Total physical activity**	
Low (<600 MET-min/week)	95 (21.1)
Moderate (600–2999 MET-min/week)	22 (4.9)
High (≥3000 MET-min/week)	334 (74.1)

MET, metabolic equivalent task. Data are n (%) or mean (standard deviation).

* Participants involved in paid work or self-employment.

**Table 2:** MET-minutes per week by domains of the GPAQ.

Domains	N = 451	
	Moderate MET-min/week	3220 (0−15,820)
Work	Vigorous MET-min/week	0 (0−2160)
	Total MET-min/week	8820 (0−35,280)
Travel	Total MET-min/week	0 (0−4320)
	Moderate MET-min/week	0(0−0)
Leisure	Vigorous MET-min/week	0 (0−0)
	Total MET-min/week	0 (0−0)
Total	Total MVPA MET-min/week	12,180 (2640−40,320)

MVPA, moderate to vigorous physical activity; MET, metabolic equivalent task. The data are median (interquartile range).

**Table 3:** Association of total MVPA MET-minutes per week with clinical measures: results of univariate analysis.

Variable	β (95% CI)	*p* Value
Age (years)	−0.02 (−0.03 to −0.01)	<0.001
Sex (female)	−0.58 (−0.78 to −0.37)	<0.001
Years of schooling	−0.036 (−0.068 to −0.004)	0.028
Occupation		
Employed *	Ref.	--
Homemaker	−1.11 (−1.34 to −0.89)	<0.001
Retired/student/unemployed	−2.50 (−2.88 to −2.11)	<0.001
Current smoking (yes)	0.56 (0.26 to 0.86)	<0.001
Current alcohol use (yes)	−0.47 (−0.71 to −0.24)	<0.001
<5 servings of fruit and vegetables/day	0.12 (−0.12 to 0.37)	0.319
Weight (kg)	−0.013 (−0.023 to −0.004)	0.007
Waist circumference (cm)	−0.025 (−0.034 to −0.016)	<0.001
Systolic blood pressure (mmHg)	−0.01 (−0.02 to −0.01)	<0.001
Diastolic blood pressure (mmHg)	−0.007 (−0.018 to 0.005)	0.248

MVPA, moderate to vigorous physical activity; MET, metabolic equivalent task; CI, confidence interval.

* Participants involved in paid work or self-employed.

**Table 4:** Association of total MVPA MET-minutes per week with clinical measures: results of multivariate analysis.

Variable	β (95% CI)	*p* Value
Model 1		
Weight (kg)	−0.011 (−0.020 to −0.002)	0.022
Systolic blood pressure (mmHg)	−0.009 (−0.016 to −0.003)	0.004
Model 2		
Waist circumference (cm)	−0.013 (−0.023 to −0.004)	0.006
Systolic blood pressure (mmHg)	−0.009 (−0.015 to −0.002)	0.007

MVPA, moderate-to-vigorous physical activity; MET, metabolic equivalent task; CI, confidence interval. β coefficients were from Generalized linear models (GLM), which were adjusted for age, sex, education, occupation, current smoking, current alcohol use, and fruit and vegetable intake.

## Data Availability

The study data is available from the first author, conditional on the ethics committee’s approval.
