# Supplementary material for: Development of a Modified Global Physical Activity Questionnaire and Its Construct Validity among Adults in Kerala, India
Source: Diabetology (Basel). Author manuscript; Available in PMC 2024 Jun 25. (PMC7615963; doi:10.3390/diabetology4020020)
Supplement: Supplementary Materials [file EMS196066-supplement-Supplementary_Materials.zip › diabetology-2403509-supplementary.pdf]

Table S1. Modified GPAQ

Physical Activity			
Next I am going to ask you about the time you spend doing different types of physical activity in a typical week. Please answer these questions even if you do not consider yourself to be a physically active person.<br>Think first about the time you spend doing work. Think of work as the things that you have to do such as paid or unpaid work, study/training, household chores, harvesting food/crops, fishing or hunting for food, seeking employment. *[Insert other examples if needed].* In answering the following questions 'vigorous-intensity activities' are activities that require hard physical effort and cause large increases in breathing or heart rate, 'moderate-intensity activities' are activities that require moderate physical effort and cause small increases in breathing or heart rate.			
**Activity at work**			
1	Does your work involve vigorous-intensity activity that causes large increases in breathing or heart rate for at least 10 minutes continuously?	Yes 1<br>No 2 *If No, go to P 3*	P1
Please note the number of days a week and time spent each day on the following activities listed below. ***Add*** *the number of days a week and time spent in a day in the total row. If the person does not do any of the activities listed below then circle "No" and go to P3.*			

2	Activity	Number of days a week	Time spent in a day	
2a	Digging	└─┘	Hours : minutes └─┴─┘ : └─┴─┘	P2
2b	Carrying, loading or stacking heavy loads such as rocks or wood	└─┘	Hours : minutes └─┴─┘ : └─┴─┘	
2c	Chopping wood or rocks with axe	└─┘	Hours : minutes └─┴─┘ : └─┴─┘	
2d	Masonry, concrete and shoveling	└─┘	Hours : minutes └─┴─┘ : └─┴─┘	

2e	Toddy tapping	└─┘	Hours : minutes └─┴─┘ : └─┴─┘	
2f	Coconut tree climbing	└─┘	Hours : minutes └─┴─┘ : └─┴─┘	
2g	Selling vegetables/fruits/milk/fish in bicycles	└─┘	Hours : minutes └─┴─┘ : └─┴─┘	
2h	Drawing water from well	└─┘	Hours : minutes └─┴─┘ : └─┴─┘	
2i	Manual grinding	└─┘	Hours : minutes └─┴─┘ : └─┴─┘	
2j	Pounding grains	└─┘	Hours : minutes └─┴─┘ : └─┴─┘	
2k	Servant maid	└─┘	Hours : minutes └─┴─┘ : └─┴─┘	
	Total	└─┘	Hours : minutes └─┴─┘ : └─┴─┘	
3	Does your work involve moderate-intensity activity that causes small increases in breathing or heart rate for at least 10 minutes continuously?	Yes 1<br>No 2 *If No, go to P 5*		P3
*Please note the number of days a week and time spent each day on the following activities listed below. **Add** the number of days a week and time spent in a day in the total row. If the person does not do any of the activities listed below then circle "No" and go to P5.*				
4	**Activity**	**Number of days a we**	**Time spent in a day**	
4a	Carrying, loading or stacking moderate loads such as bricks or stones	└─┘	Hours : minutes └─┴─┘ : └─┴─┘	

4b	Mechanic (automobile repair)	└─┘	Hours : minutes └─┴─┘ : └─┴─┘	P4
4c	Carpentry by hand	└─┘	Hours : minutes └─┴─┘ : └─┴─┘	
4d	Plumbing	└─┘	Hours : minutes └─┴─┘ : └─┴─┘	
4e	Tiles work	└─┘	Hours : minutes └─┴─┘ : └─┴─┘	
4f	Animal care: feeding, bathing, cleaning animal house, etc	└─┘	Hours : minutes └─┴─┘ : └─┴─┘	
4g	Milking cow by hand	└─┘	Hours : minutes └─┴─┘ : └─┴─┘	
4h	Gardening: watering plants, pruning, sowing seeds, cleaning, etc	└─┘	Hours : minutes └─┴─┘ : └─┴─┘	
4i	Washing clothes by hand	└─┘	Hours : minutes └─┴─┘ : └─┴─┘	
4j	Sweeping floor with a broomstick	└─┘	Hours : minutes └─┴─┘ : └─┴─┘	
4k	Mopping floor with hands	└─┘	Hours : minutes └─┴─┘ : └─┴─┘	
4l	Patient care and elderly care	└─┘	Hours : minutes └─┴─┘ : └─┴─┘	
4m	Tailoring	└─┘	Hours : minutes └─┴─┘ : └─┴─┘	

4n	Child care: dressing, bathing, grooming, feeding and occasional lifting of the child	└─┘	Hours : minutes └─┴─┘ : └─┴─┘	
	Total	└─┘	Hours : minutes └─┴─┘ : └─┴─┘	

**Travel to and from places**				
The next questions exclude the physical activities at work that you have already mentioned. Now I would like to ask you about the usual way you travel to and from places.				
5	Do you walk or use a bicycle (*pedal cycle*) for at least 10 minutes continuously to get to and from places?	Yes 1<br>No 2 *If No, go to P 7*		P5
Please note the number of days a week and time spent each day on the following activities listed below. ***Add*** *the number of days a week and time spent in a day in the total row. If the person does not do any of the activities listed below then circle "No" and go to P7.*				
	**Activity**	**Number of days a we**	**Time spent in a day**	
5a	To work	└─┘	Hours : minutes └─┴─┘ : └─┴─┘	P6
5b	To market	└─┘	Hours : minutes └─┴─┘ : └─┴─┘	
5c	To shops	└─┘	Hours : minutes └─┴─┘ : └─┴─┘	
5d	To bring children from school	└─┘	Hours : minutes └─┴─┘ : └─┴─┘	
5e	To see friends, relatives or others	└─┘	Hours : minutes └─┴─┘ : └─┴─┘	
5f	To temple, church or mosque or religious places	└─┘	Hours : minutes └─┴─┘ : └─┴─┘	

	Total	└─┘	Hours : minutes └─┴─┘ : └─┴─┘	
**Recreational activities**				
The next questions exclude the work and transport activities that you have already mentioned.<br>Now I would like to ask you about sports, fitness and recreational activities (leisure), [insert relevant terms].				
6	Do you do any vigorous-intensity sports, fitness or recreational (*leisure*) activities that cause large increases in breathing or heart rate for at least 10 minutes continuously?	Yes 1<br>No 2 *If No, go to P 9*		P7
Please note the number of days a week and time spent each day on the following activities listed below. ***Add*** *the number of days a week and time spent in a day in the total row. If the person does not do any of the activities listed below then circle "No" and go to P9.*				
	**Activity**	**Number of days a w**	**Time spent in a day**	
6a	Jogging	└─┘	Hours : minutes └─┴─┘ : └─┴─┘	P8
6b	Running	└─┘	Hours : minutes └─┴─┘ : └─┴─┘	
6c	Foot ball	└─┘	Hours : minutes └─┴─┘ : └─┴─┘	
6d	Kaleri	└─┘	Hours : minutes └─┴─┘ : └─┴─┘	
6e	Swimming	└─┘	Hours : minutes └─┴─┘ : └─┴─┘	
6f	Skipping	└─┘	Hours : minutes └─┴─┘ : └─┴─┘	
	Total	└─┘	Hours : minutes └─┴─┘ : └─┴─┘	

**Physical Activity (recreational activities)** contd.				
7	Do you do any moderate-intensity sports, fitness or recreational (*leisure*) activities that causes a small increase in breathing or heart rate such as brisk walking for at least 10 minutes continuously?	Yes<br>No	1<br>2 *If No, go to P11*	P9
Please note the number of days a week and time spent each day on the following activities listed below. ***Add*** *the number of days a week and time spent in a day in the total row. If the person does not do any of the activities listed below then circle "No" and go to P11.*				
	**Activity**	**Number of days a v**	**Time spent in a day**	
7a	Brisk walking	└─┘	Hours : minutes └┴┘ : └┴┘	P10
7b	Animal care: feeding, bathing, cleaning animal house, etc	└─┘	Hours : minutes └┴┘ : └┴┘	
7c	Gardening: watering plants, pruning, sowing seeds, cleaning, etc	└─┘	Hours : minutes └┴┘ : └┴┘	
7d	Exercise	└─┘	Hours : minutes └┴┘ : └┴┘	
7e	Volley ball	└─┘	Hours : minutes └┴┘ : └┴┘	
7f	Shuttle	└─┘	Hours : minutes └┴┘ : └┴┘	
7g	Dancing	└─┘	Hours : minutes └┴┘ : └┴┘	
	Total	└─┘	Hours : minutes └┴┘ : └┴┘	
**Sedentary behaviour**				
The following question is about sitting or reclining at work, at home, getting to and from places, or with friends including time spent [sitting at a desk, sitting with friends, travelling in car, bus, train, reading, playing cards or watching television], but do not include time spent sleeping.				
8	How much time do you usually spend sitting or reclining on a typical day?	Hours : minutes └┴┘ : └┴┘<br>hrs min s		P11<br>(a-b)
